# Conceptual Framework for Rape Survivors Diagnosed with PTSD in the North West Province of South Africa

**DOI:** 10.3390/healthcare11010127

**Published:** 2022-12-31

**Authors:** Nombulelo Veronica Sepeng, Thendo Gertie Makhado, Lufuno Makhado

**Affiliations:** 1 Department of Nursing, University of Pretoria, Tshwane 0001, South Africa; 2 Department of Advanced Nursing Sciences, University of Venda, Thohoyandou 0950, South Africa; 3 Office of the Executive Dean, Faculty of Health Sciences, University of Venda, Thohoyandou 0950, South Africa

**Keywords:** mental healthcare practitioners, PTSD guidelines, PTSD psychological management, rape survivors

## Abstract

The lack of a conceptual framework that can be utilized to manage rape survivors diagnosed with Post-Traumatic Stress Disorders presents a challenge in the North-West province. The study aims to provide a conceptual framework for managing rape survivors with PTSD in the province of the North-West using Practice-Oriented Theory and Donabedian’s Structure Process Outcome Model Features. The research was conducted using an explanatory, sequential and mixed-methods approach. Additionally, used was the descriptive and explorative programme evaluation design. The results of the study demonstrated the significance of PTSD assessment before management interventions for rape survivors. The study findings outlined and designed a framework to assess and manage PTSD among rape survivors consulting at Thuthuzela Care Centre and those referred to hospitals for further management. The Practice-Oriented theory by Dickoff, James and Wiedenbach, and the Structure Process Outcome model by Donabedian served as points of reference for the development of the conceptual framework. The study is limited to North-West provincial healthcare facilities and Thuthuzela care centres (TCCs), however, it highlights the lack of a conceptual framework pertaining to the psychological management of PTSD rape survivors in the province and South Africa.

## 1. Introduction

Rape is a global issue that affects everyone everywhere. Compared to other countries, South Africa has a high rate of rape. According to the South African Police Service [1], between April and June 2022, 9516 rape cases were opened. While other provinces had a decrease in rapes, North West Province had a higher increase in rapes than other provinces in South Africa [1]. There is a concern that rape affects the mental health of survivors, resulting in Post-Traumatic Stress Disorder (PTSD) [2]. According to Sepeng and Makhado [3], 74.5% of rape survivors living in the North West province of South Africa have PTSD. In South Africa, rape survivors are primarily managed in a one-stop clinic called Thuthuzela Care Centre (TCC). The TCCs were established to provide multidisciplinary trauma-related management through a variety of healthcare professionals, including professional nurses, social workers, psychologists and medical doctors. However, some TCCs do not have all the staff members, particularly the psychologists and medical doctors. Therefore, social workers and nurses working within TCCs are the ones responsible for providing psychological support to rape survivors [4].

Despite this, Olckers [5] explained that in South Africa, social workers are not trained to use diagnostic and statistical manual (DSM) for diagnosing mental health disorders, including how to provide mental health care management using cognitive behavioural therapy (CBT) in undergraduate level as compared to psychologists. The author, Olckers [6], further explained that social workers with clinical master’s degrees are taught how to use the DSM to diagnose mental health disorders such as PTSD and anxiety-related disorders and manage mental health disorders. This also gave birth to controversies within the South African Society of Psychiatrists (SASOP) guideline for social workers to diagnose and manage mental health disorders as this is not the scope of practice in South Africa [6]. In this aspect, clinical psychologist and psychiatrist have deemed the champions for assessing and providing mental health care amongst patients [7].

Since 1954, psychiatric nurse specialists have been providing psychotherapy in public health care facilities [8]. However, in South Africa, the position of advanced practice psychiatric nurse must be clearly defined, accepted, and recognized by regulatory bodies and mental health practitioners [9]. This is true given that specialists such as psychiatrists and psychologists are not always available at many public health clinics. This makes the psychiatric nurses and psychiatric nurse specialists frequently expected to perform tasks that psychiatrists and psychologists would typically perform [10]. Given the limited resources available in Low and Middle-Income Countries such as South Africa, task-shifting mental health care delivery to nonspecialist providers is recommended [11,12]. In support of this, a protocol paper of the study will be conducted in Cape Town on task-shifting advocates for training nurses with mental health on the use of CBT with the support of psychologists [13]. Given this issue of task shifting the provision of mental health to nurses, Chetty and Hoque [9] conducted a study on nurse-facilitated cognitive group intervention among patients diagnosed with mild depression. In their study, they found that the symptoms of depression were diminished after 12 weeks of therapy. Hence, they proposed the need for task-shifting mental health care management to psychiatric nurses in LMIC, such as in South Africa, wherein there is a shortage of psychologists and psychiatrists [9].

As such, a conceptual framework (CF) for the psychological management of rape survivors with PTSD must be developed. In agreement, Mansfield [14] and Hoeffer [15] highlight how crucial it is to have a precise CF before creating guidelines for treatment in psychiatric-mental healthcare.

Models created by Gatchel, Peng Peters, Fuchs and Turk [16]; Sanders, Harden, and Vicente [17]; and Gatchel [18] are among those that have been used in the conceptualization of a framework for PTSD and pain treatment [19]. However, the current study singles out the Practice-Oriented Theory (POT) by Dickoff, James and Wiedenbach [20] and the Structure Process Outcome (SPO) model by Donabedian [21]. The two were determined to possess the crucial characteristics required to conceptualize a framework that can address the current study’s empirical findings [3,22,23]. Therefore, a CF was developed using SPO and POT features as a conceptual basis for assessment and management of adult women rape survivors diagnosed with PTSD at TCCs and public mental health facilities in the North-West province

### 1.1. Conceptual Framework

Donabedian’s [21] SPO model was adopted as the prototype because of its features, which include the process, structure and outcome. The POT model was selected as the second prototype to be incorporated into the SPO characteristics to build a CF for evaluating and treating rape survivors with PTSD in TCC and public mental hospitals in the North-West Province.

The features of POT include the agent, recipients, and other stakeholders, context as well as procedure, dynamic and terminus [20]. An SPO model incorporates the involvement of an agent, recipients and stakeholders within the structure, process to achieve the desired outcomes [21]. The dynamic has to be examined independently [19], whereas the terminus has to be integrated into the outcome since they both mean the same thing [20,21].

### 1.2. Structure

According to Donabedian [21], the structure is a setting in which it is necessary to have the structural resources, requisite equipment and personnel to provide the required management. The agent describes a person or item in the structure that is in charge of carrying out an activity and is embedded within the structure. Based on the conceptualized findings of this study, multidisciplinary healthcare practitioners should perform psychological management activities for PTSD. Mental healthcare practitioners must be trained in assessing, diagnosing and managing PTSD. The recipient, referred to as the person or thing that receives service from the agent, is also embedded in the structure [20]. In this study, all recipients were adult women rape survivors who were diagnosed with PTSD. These adult women rape survivors should either be married or single. According to Dickoff et al. [20], non-professional stakeholders may be considered supporters of recipients undergoing treatment. The CF should identify relevant stakeholders to support survivors who are undergoing treatment.

### 1.3. Process

According to Donabedian [21], the process follows the structure and is defined as guiding principles. The process includes rules, techniques, protocols, and routine governing activities to be undertaken to achieve the outcome. As described in the Diagnostic Statistical Manual-5 (DSM-5), this process follows the guiding rules and protocols used for assessing Acute Stress Disorders. The DSM-5 for Acute Stress Disorders defines the criteria for managing and preventing PTSD. The psychological management approaches are then put into practice to diagnose and manage PTSD accurately. It specifies how many further appointments a rape survivor must make to finish treatment and reach the intended result or conclusion [24]. However, the necessity for dynamic sources of power between activities exists. In the CF, the sources of power allow an agent to carry out the suggested process (routines governing activities, protocol, techniques and guiding rules) to improve the success of the results [20].

### 1.4. Outcome

According to Donabedian [21], the activity’s final output is the result or results. Similarly, Dickoff et al. [20] describe the endpoint as the outcome of the agent’s actions to better results. Therefore, while the agent is giving psychological management, it is necessary to describe the intended outcome.

### 1.5. Context

The CF of this study was expanded to include the context, which is defined as the setting that enables the agent to carry out activities requested by the recipient [20]. Meaning the environment in which the rape survivors with PTSD will receive care must be clearly defined.

## 2. Methods

### 2.1. Study Design

An explanatory sequential mixed-method research design was used for this study. An explanatory-sequential approach is a sequential approach used when the researcher wants to supplement quantitative findings with qualitative data [25]. Thus, qualitative data are used to interpret and clarify quantitative data analysis results [25]. This two-phase approach is especially beneficial for a researcher who wants to explain the findings from the first phase of the study using qualitative data collected during Phase 2 [25].

### 2.2. Population and Sampling

Adult rape women survivors and mental healthcare professionals who resided in the North West province at the time of data collection. Purposive sampling was used to select rape survivors and mental health care practitioners. Adult rape women survivors agreed to participate in a quantitative phase were 98, and 21 mental health care practitioners agreed to participate in a qualitative phase.

### 2.3. Data Collection Procedure

In phase 1 of the study, data were collected using PDS-5 developed to determine the prevalence of PTSD among adult rape women survivors [26]. The PDS-5 is a 24-item self-report measure that assesses the severity of PTSD symptoms in the previous month using DSM-5 criteria [26]. In our study, the 1st author interviewed the respondents using the PDS-5 scale translated into their local language. The PDS-5 begins with two trauma screen questions designed to assess the respondent’s trauma history and identify the traumatic event causing the respondent the most distress [26]. Twenty questions assess the presence and severity of PTSD symptoms concerning the index trauma; symptom questions are based on the DSM-5 symptom clusters intrusion (Items 1–5), avoidance (Items 6–7), mood and cognition changes (Items 8–14), and arousal and hyperreactivity (Items 15–18), (Items 15–20) [26]. The symptom items are rated on a 5-point frequency and severity scale ranging from 0 (not at all) to 4 (6 or more times per week/severe) [26]. The Cronbach’s alpha reliability of PDS-5 of this population was at 0.89 [3]. Comprehensive Medical Care Management (CMCM) survey developed by Patel et al. [27] was also used to interview rape survivors regarding medical care, acute mental health and chronic mental health care management received in TCCs. Adult rape survivors were requested to respond by indicating “Yes” or “No”. The Cronbach’s alpha reliability scores of the CMCM in this population were 0.81 [22].

Focus group discussions were conducted with mental health care practitioners after analysing quantitative data from the first phase. The first question during focus group discussions with mental health care practitioners was their perceptions regarding mental health care services for adult rape survivors diagnosed with PTSD consulting in TCCs. The other questions included what could be the barriers to providing psychological management for rape survivors diagnosed with PTSD, and which assessment measures must be used to diagnose PTSD among rape women survivors? Followed by which interventions must be used for rape survivors? Additionally, which mental health care practitioners are eligible to provide care for adult rape women survivors diagnosed with PTSD? The results of the two phases were published as stand-alone quantitative and qualitative papers [3,22,23]. In this paper, we are reporting the integrated findings of phase 1 and 2 to develop a conceptual framework using SPO and POT features as a conceptual basis for rape survivors with PTSD who consult at TCCs and public mental health facilities in the North-West province. The researchers adhered to Lincoln and Guba’s (1985) five criteria of trustworthiness: credibility, conformability, neutrality, dependability, and transferability.

### 2.4. Data Analysis

The data for the quantitative phase were analysed independently using SPSS, and the results of qualitative data were analysed independently following thematic analysis, see published papers by Sepeng and Makhado [3], Sepeng and Makhado [22] and Sepeng, Makhado and Sehularo [23]. In this paper we are presenting the integrated findings of quantitative and qualitative phase to develop a CF for the psychological management of PTSD for rape survivors consulting in TCC and when they are referred to a public provincial/district hospital for additional treatment. Data analysis for this paper was performed following inferences. In mixed methods research, inferences are defined as “conclusions or interpretations drawn from a study’s separate quantitative and qualitative strands as well as from across the quantitative and qualitative strands” [28]. In mixed method designs, data inferences would be better achieved by collecting data from multiple sources using different methods [28]. The inferences were then used to answer the main objective of this paper, which the researcher has combined, contrasted, compared, and interpreted findings from the qualitative and quantitative stages.

### 2.5. Ethical Considerations

The Human Sciences Research Ethical Committee at North-West University (NWU) granted consent for the study (ethics number NWU-0477-17-A9). The North West Province Department of Health was also approached for approval and was granted. Participants studies voluntarily provided a written consent form to conduct the study. Data from adult rape women survivors were collected in Thuthuzela care centres to refer them for further management should they break down during the interviews. Data collection for mental health care practitioners was carried out in mental health care institutions in their spare time to avoid the routine work of caring for their patients.

## 3. Results and Discussion

### 3.1. Structure

**Figure 1:**
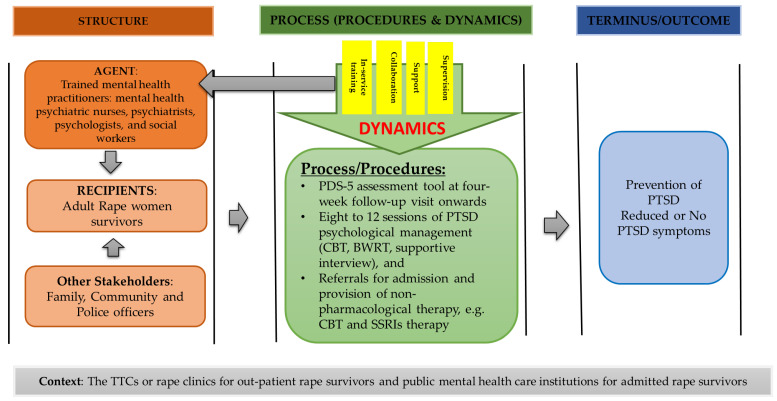
A conceptual framework for assessment and management of adult rape women survivors diagnosed with PTSD in TCCs and public mental health care facilities.

The CF structure represents the demographic data of the participants in the qualitative and quantitative phases of the study, including the suggested key stakeholders that may offer support to rape survivors undergoing care in the health care system. Therefore, the recipients of this CF, the respondents that were interviewed during the quantitative phase, and they included adult rape women survivors, consulted in TCCs at the time of data collection. Adult rape women survivors were aged 18 years and above. Mostly believed in Christianity; some were single, married, and received care at any time they reported rape. Their level of education differed in terms of being less educated, and some were educated. The agents of the CF included the participants that were interviewed during the qualitative phase and included practitioners in the mental healthcare field, such as psychiatrists, psychologists, nurses, doctors and social workers. The agents of this CF are mental healthcare professionals who are required to do PTSD assessment and management with a variety of shared tasks in accordance with their scope of practice [29]. Other key stakeholders that mental healthcare practitioners suggested during qualitative phase of the study to support for adult rape women survivors receiving psychological management for PTSD within the health care system were people of the community, family members and SA police officers. The involvement of these stakeholders, regarded as social support in the CF, can only be carried out in accordance with the rape survivor’s consent. This is implemented to avoid violating the human rights and dignity of survivors [30]. Additionally, Gordon [31] and Welch and Mason [32] emphasized that the involvement of support structures for survivors dealing with the after-effects of trauma is needed because they help survivors cope with the event and, in turn, indirectly minimize PTSD symptoms. Hence, the stakeholders are included in the developed CF in Figure 1 mainly to support adult rape women survivors undergoing treatment for PTSD.

### 3.2. Process and Outcome

The process that was outlined in this framework was the process that must be carried out by mental health care practitioners when caring for the rape survivor. The study’s results in the quantitative phase revealed that the PDS-5 questionnaire might be used to diagnose PTSD among adult rape women survivors consulting in the health care system from six weeks onwards post-rape experiences. In support of this, a study conducted by Sepeng and Makhado [3] revealed that about 74.5% of rape survivors were diagnosed with PTSD post-rape experiences within six weeks using the PDS-5 tool. Mental health care practitioners further supported the use of PDS-5 in a qualitative phase. They have indicated that it is procedurally correct to measure adult women rape survivors’ PTSD using the PCL-s or PDS-5 tools. Mental health care said during a focus group:

*“Mental health care practitioners working in TCCs must book rape survivors for follow-up at six weeks to assess them for PTSD symptoms using tools that are linked to statistical diagnostic manual, for example PCL-s and PDS-5…”*

(identified as a female, psychologist, 20 years of service).

*“…as such the same diagnostic tools must be used to assess them whey are admitted in psychiatric mental health care institutions”*

(identified as a male, psychiatrist, 28 years of service).

These diagnostic instruments, PCL-5 or PDS-5 tools are reliable techniques for identifying PTSD in adult rape women survivors [26,33].

The results of the study in the quantitative phase revealed that adult rape women survivors were not scheduled for assessment and management of long-term mental health disorders in TCCs post-rape experiences [22]. The reasons for not being scheduled for assessment and management for long term management were not explored among adult rape women survivors because of the design used was quantitative in nature. However, during the qualitative phase, mental health care practitioners perceived that it could be due to “*lack of human resources in other TCCs, a lack of experienced staff to schedule rape women survivors for assessment and management of PTSD and rape survivors not attending follow-up care due to personal reasons such as having to travel long distances to reach TCCs and lack of disclosure*” (identified as male, social worker, ten years of service and identified as female, psychiatric nurse 19 years of service). In support of this, Davis et al. (2008:218) state that several barriers to care have been identified in the literature as potentially preventing PTSD patients from receiving treatment, such as lack of human resources in LMIC, the experience of self-blame by rape survivors and stigma attached to it.

During a qualitative phase, mental health care practitioners were asked which mental health care interventions can be used to manage rape survivors diagnosed with PTSD. In their response, they indicated that:

“…it is procedurally correct for mental health care practitioners to manage adult rape women survivors diagnosed with PTSD through using Cognitive Behavioural Therapy (CBT), Eye Movement Desensitization and Reprocessing Therapy (EMDR), exposure therapy, Cognitive Processing Therapy (CPT) or supportive counselling for effective management or remission of symptoms at least for 12 weeks”

[23].

These results are consistent with the studies conducted by Foa and McLean [34], Watts [35] and Ochberg [36] that the use of cognitive behavioural therapy (CBT), eye movement desensitization and reprocessing therapy (EMDR), exposure therapy, cognitive processing therapy (CPT), supportive counselling, or as well as for effective management or remission of PTSD symptoms. These interventions require at least 8 to 12 sessions with the client diagnosed with PTSD [37].

Furthermore, mental health care practitioners suggested that

*“The is a need to give rape survivors diagnosed with PTSD and admitted at the hospital selective serotherapy (SSRIs)”*

(identified male, psychiatrist, nine years of service).

In support of Kirkpatrick and Heller [38] suggest that SSRIs must be given to hospitalized patients for at least 30 days. However, the course of treatment must last for a full year and include outpatient follow-up [38]. Despite this, Mclean and Foa [39] stated that existing methods often require a relatively high investment of resources unavailable to all affected individuals. This is especially true for people with low socioeconomic status and those living in developing countries, where the government healthcare service and welfare infrastructure may be underdeveloped, inaccessible, or expensive [40,41]. However, even when such treatments are made available, other factors such as shame and stigma may prevent trauma victims from seeking help [40,41]. Furthermore, task shifting must be considered a desirable strategy that can be used to address issues pertaining to human resources to provide mental health care services [42].

Mental health care practitioners also stated that brain working recursive therapy (BWRT), which was developed by Watts [35], must be used to treat adult women rape survivors diagnosed with PTSD. One of the mental health care practitioners with others nodding their heads in support of what she saying during a focus group discussion indicated that:

*“Have at least one or two sessions of Brain Working Recursive Therapy (BWRT) with rape survivors diagnosed with PTSD”*

(identified as a female, psychologist, 5 years of service).

However, Marsay [43] stated that given the growing popularity of the BWRT method, it is critical to collect data on its effectiveness. Suppose BWRT as a one-session treatment produces results comparable to treatment as usual. In that case, there is a strong and compelling case for BWRT to be established as a runner-up for future trauma treatment research [43]. Therefore, in this framework, we are proposing the adoption of these mental health interventions and urge South African government to allocate resources needed to care mental health of rape survivors diagnosed with PTSD.

During the qualitative phase, mental health care practitioners stated their dynamic sources of power between activities they needed to carry out the process of assessing and managing adult rape women survivors diagnosed with PTSD. They have suggested that:

*“There is a need for experienced mental health care practitioners to support and supervise nurses and social workers caring for rape survivors on how to assess, diagnose and manage rape survivors with PTSD”*

(Identified female, psychiatric nurse, six years of service and identified female, psychologist, nine years of service).

*“…also, mental health care practitioners working in public health care institutions should avail themselves to work in collaboration with TCCs staff members. For example, they may have a special day of working with TCCs staff to provide support and supervising them on how to assess and manage rape women survivors for PTSD when scheduled for follow-care until such time they are competent to assess and manage rape survivors on their own”*

(Identified female, psychiatric nurse, 14 years of service and identified female, psychiatric nurse, seven years of service).

These findings are supported by Wangamati et al. [44] and Moylan and Lindhorst [45] by emphasizing the need to encourage multidisciplinary teams of mental healthcare professionals to work together to offer better quality care when caring for their patients. If this practice of allowing mental health care practitioners working in mental health care institutions to support and supervise staff working in TCCs is implemented, it will assist the government in achieving the promotion of access to mental health care services, particularly for rape survivors diagnosed with PTSD.

Furthermore, mental health care practitioners during a focus group discussion also suggested that:

*“There is a need for a psychologist in public mental health care institutions to provide in-service training for nurses and social workers working in TCCs on how to assess and manage rape survivors diagnosed with PTSD”*

(identified female, social worker, 11 years of service).

*“Also, the in-service training must be done for mental health care practitioners responsible for assessing and managing rape survivors diagnosed with PTSD in case there are new treatment modalities that can be used to manage rape survivors diagnosed with PTSD, including new assessment scales when adjusted according to the new diagnostic and statistical manual of mental health disorders”*

(identified male, psychologist, eight years of service).

In support of this, Abrahams and Gevers [46] found that mental healthcare professionals require ongoing in-service training to provide quality psychological management of PTSD among rape survivors. Whilst supervising and conducting in-service training for nurses and social workers working in TCCs, rape survivors can be referred to mental health care institutions for assessment and management of PTSD. Additionally, the department of health should appoint nurses that specialized in psychiatry in TCCs in LMIC to assess and manage rape survivors diagnosed with PTSD subject and make sure they receive support, supervision and in-service training to promote the quality care needed by rape survivors. Thus, the importance of carrying out the process of assessing adult rape women survivors as outlined in this CF is to reach the outcome of diminishing or reducing PTSD symptoms among adult rape women survivors.

### 3.3. Context

Mental health care practitioners suggested TCCs and mental health care institutions context to manage adult rape women survivors diagnosed with PTSD. Mental health care practitioners stated interventions such as CBT might be carried out in TCCs for outpatient adult rape women survivors. The findings of this study in CF support previous studies that have advocated for decentralization of the management of PTSD in rape care clinics [46]. Furthermore, interventions such as CBT and SSRIs may be carried out among adult rape women survivors admitted in public mental health care institutions and maybe continued in outpatient departments of mental health care institutions when the survivor is discharged. Thus, achieving the goal of managing rape survivors diagnosed with PTSD in a resource-constrained country such as South Africa. The CF shown in Figure 1 is created to direct the process of assessing and managing adult women rape survivors diagnosed with PTSD at TCCs and public mental health facilities.

## 4. Practical Implications of the Study

The CF developed can help with beginning to assess and manage adult rape women survivors diagnosed with PTSD in TCCs in South Africa, working in collaboration with public mental health care institutions for those in need of admission. This practice will help developing countries such as the South African government promote access to mental health care services among those in need. Using this CF can help decentralize mental health care management, task shifting and collaborative work between mental health care practitioners and inform the curriculum of other health care professionals to educate them on how to care for adult women rape survivors in resource-constrained countries such as South Africa.

## 5. Limitations

It is not possible to use CF for the psychological treatment of other traumatic situations because it was developed primarily on the experiences of adult women rape survivors. It can, however, act as the cornerstone for such advancement.

## 6. Conclusions and Recommendations

A CF for evaluating and treating adult women rape the study developed survivors who have been diagnosed with PTSD. The framework is essential because it serves as the foundation and provides for the psychological management of adult women rape survivors diagnosed with PTSD in LMICs such as South Africa. Therefore, this study suggests that the CF be adopted for assessing and treating adult women rape survivors diagnosed with PTSD. The developed CF also advocated for task-shifting and decentralising mental health care services to primary health care facilities. The CF also indicated the importance of allowing various healthcare professionals to work together when providing mental healthcare services for rape survivors diagnosed with PTSD despite working in tertiary and primary healthcare facilities. Thus, promoting equal access to mental health care services, collaboration, training opportunities and support from one another.

## Data Availability

The data presented in this study are available on request from the corresponding author. The data are not publicly available due to the ethical clearance conditions and given the sensitivity of the study.
